# Intraoperative Imaging and Optical Visualization Techniques for Brain Tumor Resection: A Narrative Review

**DOI:** 10.3390/cancers15194890

**Published:** 2023-10-09

**Authors:** Othman Bin-Alamer, Hussam Abou-Al-Shaar, Zachary C. Gersey, Sakibul Huq, Justiss A. Kallos, David J. McCarthy, Jeffery R. Head, Edward Andrews, Xiaoran Zhang, Constantinos G. Hadjipanayis

**Affiliations:** 1 Center for Image-Guided Neurosurgery, University of Pittsburgh Medical Center, Pittsburgh, PA 15213, USA; binalameroa@upmc.edu (O.B.-A.); aboualshaarh@upmc.edu (H.A.-A.-S.); gerseyzc@upmc.edu (Z.C.G.); huqs5@upmc.edu (S.H.); kallosja@upmc.edu (J.A.K.); mccarthydj2@upmc.edu (D.J.M.); headjr@upmc.edu (J.R.H.); andrewse2@upmc.edu (E.A.); zelbest@gmail.com (X.Z.); 2 Department of Neurological Surgery, University of Pittsburgh Medical Center, Pittsburgh, PA 15213, USA

**Keywords:** intraoperative imaging, brain tumor surgery, advanced visualization techniques

## Abstract

**Simple Summary:**

Brain tumors are difficult to treat, and surgeons need the best tools to safely remove them. This review looks at the various technologies that help surgeons see tumors more clearly during surgery. These technologies range from special microscopes and exoscopes to advanced imaging like handheld molecular diagnostic tools. While these tools have made surgery safer and more effective, they are not without challenges, such as complex usage and interpretation. Our aim is to present an overview of these technologies, discuss their pros and cons, and look at the future, where artificial intelligence and virtual reality could make these surgeries even more precise. This research could guide future innovations that improve patient outcomes.

**Abstract:**

Advancements in intraoperative visualization and imaging techniques are increasingly central to the success and safety of brain tumor surgery, leading to transformative improvements in patient outcomes. This comprehensive review intricately describes the evolution of conventional and emerging technologies for intraoperative imaging, encompassing the surgical microscope, exoscope, Raman spectroscopy, confocal microscopy, fluorescence-guided surgery, intraoperative ultrasound, magnetic resonance imaging, and computed tomography. We detail how each of these imaging modalities contributes uniquely to the precision, safety, and efficacy of neurosurgical procedures. Despite their substantial benefits, these technologies share common challenges, including difficulties in image interpretation and steep learning curves. Looking forward, innovations in this field are poised to incorporate artificial intelligence, integrated multimodal imaging approaches, and augmented and virtual reality technologies. This rapidly evolving landscape represents fertile ground for future research and technological development, aiming to further elevate surgical precision, safety, and, most critically, patient outcomes in the management of brain tumors.

## 1. Introduction

Brain tumor imaging has been a longstanding area of multidisciplinary interest due to the unique demands and complexities of managing this patient population [1,2]. While diagnostic imaging techniques for brain tumors are relatively well characterized, real-time intraoperative imaging remains an area of active need and interest; this is particularly relevant in gliomas and other intra-axial tumors in which safe, maximal resection remains a mainstay of treatment [2,3,4].

Surgical resection relies heavily on the surgeon’s ability to delineate the tumor from the surrounding normal parenchyma to achieve maximal safe resection [5,6]. Such precision hinges on effective intraoperative imaging which provides real-time guidance during resection and helps to confirm whether the desired extent of resection has been achieved [5,7]. This need is underscored by a robust body of evidence describing the relationship between the extent of resection and patient outcomes, including progression-free and overall survival, particularly in surgery for gliomas [6,8,9,10,11].

To date, conventional intraoperative imaging modalities such as intraoperative ultrasound (iUS), magnetic resonance imaging (iMRI), and computed tomography (iCT) have played a significant role in guiding brain tumor resection [1,5]. Recent advancements in the capabilities of nanomaterials have shown promise in both T1-MRI and photodynamic therapy [12,13]. Cellular/subcellular imaging techniques like Raman-based spectroscopy provide excellent diagnostic capabilities, but are often too focused to evaluate the completeness of resection comprehensively. On the other hand, supracellular imaging modalities like iCT, iMRI, and iUS offer a more expansive view essential for assessing resection boundaries, but may lack the diagnostic specificity offered by cellular/subcellular imaging [5,14,15]. Recent advancements in optical imaging techniques may provide exciting new avenues for intraoperative visualization; these include exoscope-based visualization, fluorescence-guided surgery (FGS), confocal imaging, and Raman-based technologies [4,7,16,17]. An integrative approach may involve the combination of cellular/subcellular imaging for diagnostic specificity with supracellular imaging techniques for a broader assessment of resection. This combination could potentially enhance the precision and effectiveness of brain tumor surgeries. These technologies are a promising addition to the surgical armamentarium in the era of molecularly informed precision medicine for brain tumor patients.

This review aims to comprehensively explore FDA-approved or clinically accepted optical imaging techniques, focusing on their strengths, limitations, and potential applications in brain tumor surgery. We have intentionally limited the scope of our review to technologies that are currently available for clinical use, thereby excluding experimental and preclinical studies. We describe the operational principles of these modalities, their unique attributes, and challenges they address in the surgical setting. We also discuss future directions and innovations in this rapidly evolving field.

## 2. Optical Visualization Techniques for Brain Tumor Resection

### 2.1. A Historical Lens on the Evolution of Optical Neurosurgical Oncology

**Figure 1:**
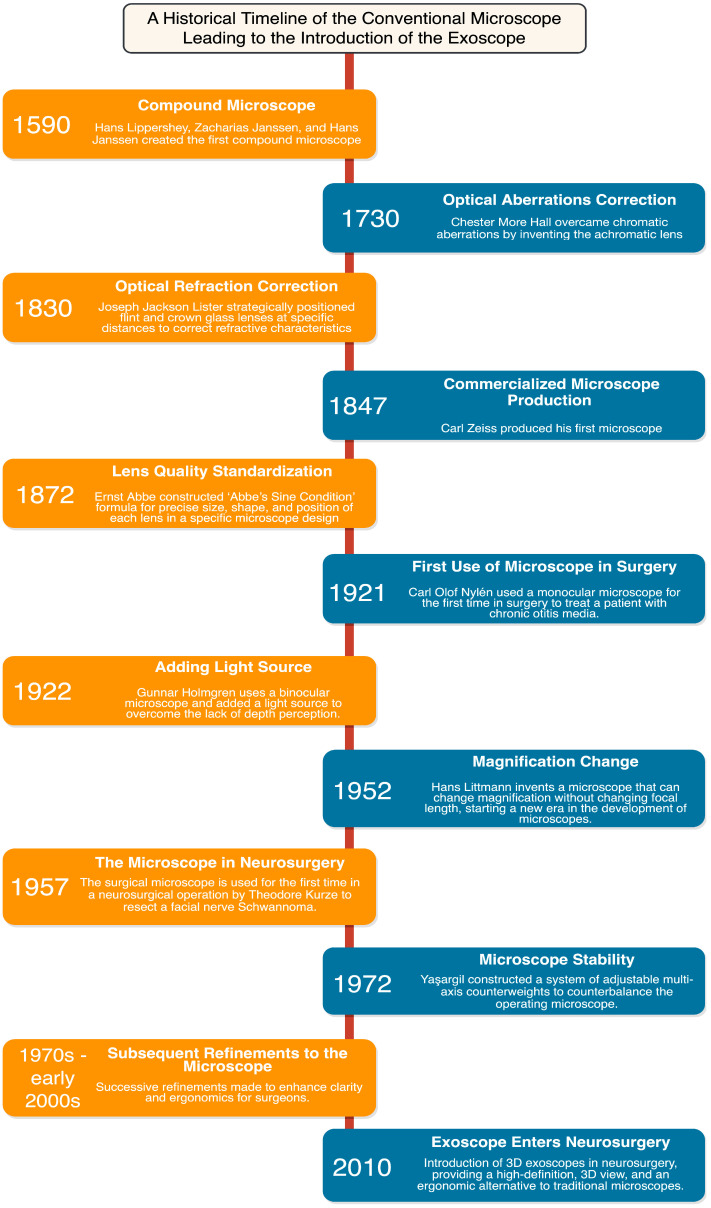
A Historical Timeline of the Conventional Microscope Leading to the Introduction of the Exoscope.

The development of the operating microscope and subsequent evolution of microsurgical techniques is a journey that spans centuries, characterized by human ingenuity and punctuated by scientific breakthroughs (Figure 1) [18]. Initial strides in these technologies occurred in the 19th century, when individuals such as Chester More Hall and Joseph Jackson Lister sought to correct optical aberrations, which improved image clarity and magnification [18,19]. Carl Friedrich Zeiss began to specialize in microscope manufacturing during the mid-19th century and, together with Ernst Carl Abbe, they contributed to the standardization and production of high-quality microscopes [18,20].

By the 20th century, the microscope had become an integral component of many surgical procedures, demonstrated first by Carl Olof Nylén, who utilized a monocular microscope for labyrinthine fistula surgery in 1921 [21]. In 1938, a heavy tripod with counterweights was introduced, which improved stability during high magnification [18]. In the late 1940s, the binocular surgical microscope was introduced by Richard A. Perritt; this binocular technology allowed surgeons to have enhanced depth perception and adjustable magnification [18,19,22]. In 1952, Hans Littmann developed the Zeiss-Opton microscope, which could change magnification without altering the focal length. By 1953, the “Zeiss OPMI 1” operating microscope was manufactured, which offered enhanced stability, user-friendly operation, and improved coaxial lighting [19,22,23]. This period marks the integration of microscopes in neurosurgery, first by Theodore Kurze in Los Angeles, with adaptations for ophthalmological surgery by Heinrich Harms, Günter Mackensen, and Jose Ignacio Barraquer [18,19,23]. As Kurze advanced his work, Raymond M. P. Donaghy simultaneously began enhancing the operating microscope on the opposite coast in Vermont [24]. Additional innovations introduced in 1956, such as axial illumination and foot-operated controls, were complemented by the introduction of a mouth switch, which collectively facilitated the ease of microscope use in surgery [19,23]. These advancements led to an increase in the use and development of operative optical devices for various surgical procedures, including craniotomies and brain tumors resections.

### 2.2. Conventional Operating Microscope

Brain tumor resection is a delicate and challenging surgical procedure that demands optimal visualization and precision [9,21,22]. The operating microscope found its way into the neurosurgical operating room in 1957, where Theodore Kurze utilized it to resect a schwannoma from a young patient at the University of Southern California in Los Angeles (Figure 1) [19]. Following this success, neurosurgeons worldwide quickly recognized the microscope’s potential and started implementing and refining its use during brain tumor resections [18,25].

The integration of the operating microscope into neurosurgical practice resulted in unprecedented high-definition visualization and magnification of the surgical field, which facilitated the differentiation between normal and abnormal tissue (Table 1) [26]. This accurate delineation is of utmost importance, as it enables the surgeon to perform maximal safe resection while preserving vital structures, therefore reducing postoperative neurological deficits and morbidity [11,27,28,29]. Studies have consistently demonstrated that a greater extent of resection translates into longer progression-free survival and overall survival in patients with malignant brain tumors, particularly gliomas [5,9,11,27,28,29,30].

Key advancements include the adaptation of beam splitter technology, the addition of surgeon armrests and patient headrests, and the development of counterweights to balance the operating microscope [18,19,31]. The operating microscope has paved the way for various complementary technologies such as the use of fluorescence-guided surgery, facilitated by modules such as the FL-400 and FL-800 [18]. Furthermore, the ability to record surgical procedures in high-definition quality offers a valuable tool for teaching, consultation, self-improvement, and future research, ultimately contributing to continual enhancements in tumor resection strategies [18].

Despite the numerous advancements and the clear benefits, the use of the operating microscope in brain tumor resection is not without challenges [32]. One main limitation is the restricted field of view, which can pose difficulties when operating on lesions located in complex anatomical regions or deep within the brain tissue [26,33,34]. Additionally, despite counterweight systems and electromagnetic brakes, the bulk and weight of operating microscopes can still limit maneuverability, particularly in long procedures [7,34,35]. The eyepiece-based viewing system, while offering excellent visualization, has limited magnification and illumination and may lead to operator fatigue over time [18]. Ergonomics are another significant consideration, including the need for challenging neck and back positioning that can impact surgeon fatigue during longer surgeries; recent work has also elucidated the ramifications of intraoperative ergonomics on surgeon health and career longevity. These challenges set the stage for the development of newer technologies discussed in subsequent sections.

### 2.3. Exoscope

**Figure 2:**
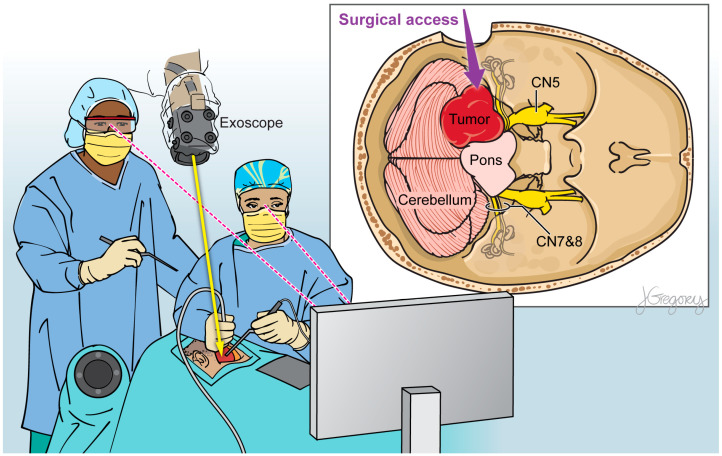
Illustration of a 3D exoscope view detailing the relevant anatomical structures during brain tumor resection.

Within the ever-evolving domain of neurosurgical optics, the exoscope has emerged as an exciting improvement upon the conventional operating microscope (Figure 1; Table 1) [17]. Its operating principle involves the use of a camera system positioned alongside the surgeon that provides two- or three-dimensional, high-resolution imaging on a heads-up display monitor placed in front of the surgeon (Figure 2) [4,36,37,38,39]. The exoscope may offer improvements in visual acuity and operative workflow compared to traditional binocular surgical microscopes due to greater magnification, illumination, and depth of field perception [40,41,42].

A recent systematic review compared the exoscope to the traditional operating microscope and explored their applications to neurosurgery; across all papers, the exoscope’s video image quality, three-dimensional visualization, and surgical field illuminated were found to be comparable or even superior to those provided by the microscope [17]. The exoscope was found to nicely facilitate the visualization of critical neurovascular structures, cerebral parenchyma vs. tumor, and operative instruments across both superficial and deep operative fields [40,41,42]. Neuro-oncology has been a key application of the exoscope at leading centers [33,37,43].

The exoscope has also provide significant improvements in surgeon ergonomics by promoting a more relaxed posture and alleviating the physical strain associated with the use of conventional operating microscopes [33,44]. This is particularly true when two surgeons are performing microsurgery together; while this is often a cumbersome exercise with the conventional operating microscope, it is far more natural with the exoscope. 

Recent exoscopes incorporate features such as light filters for 5-aminolevulinic acid (5-ALA), fluorescein, indocyanine green (ICG) video-angiography, and adjustable operative settings [4,40,45]. A recent study found that the exoscope provided superior visualization under blue light and required fewer switches between blue and white light (median = 10), thereby enhancing the surgical process compared to the traditional operating microscope (median = 14) [40]. This amalgamation of functionalities bolsters surgical precision and safety, while the capacity for collective visualization encourages improved intraoperative communication and surgical workflow. However, their practical effectiveness needs more systematic and comprehensive assessment.

Several studies examined the extent of resection attained while using the exoscope and have shown an average extent of resection of up to 95% and a rate of complete resection ranging from 65% to 80% for various brain tumor types [37,46,47,48,49]. However, it is essential to consider that these figures may vary, and more expansive, rigorously designed studies are needed to assess the exoscope’s true impact on the extent of resection, along with other important aspects such as, safety, ease of use, postoperative complications, patient survival, and surgeon comfort. 

Despite these advantages, several drawbacks have been reported. Transitioning from direct visualization to monitor-based viewing with the exoscope presents a learning curve for surgeons accustomed to traditional microscopes [36,50]. A recent systematic review conducted by Montemurro et al. examined 21 clinical series involving a total of 891 patients [17]. Among these cases, 5.8% (52 instances) opted to transition from using the exoscope to a traditional operating microscope during surgery due to the steep learning curve associated with exoscope technology. Financial constraints are also a significant limitation to the widespread adoption of the exoscope [51]. The acquisition and maintenance costs associated with exoscope technology are substantial and may not be feasible for all healthcare settings, especially where a microscope is already available. 

The exoscope holds significant promise for further development. It is speculated that future versions might incorporate artificial intelligence and machine learning algorithms for automated delineation of tumor boundaries and critical neurovascular structures, which could facilitate improved surgical safety and efficiency [52]. In sum the exoscope represents a notable development in neurosurgical procedures with significant implications for neuro-oncology; it promises to be an area of significant ongoing research and development in the years ahead [33].

### 2.4. Fluorescence-Guided Neurosurgery

Fluorescence-guided surgery (FGS) is an exciting innovation in neuro-oncology that facilitates augmented visualization of brain tumor tissue by inducing selective fluorescence in tumor cells [4]. FGS has been seamlessly integrated into existing optical modalities, including loupes, microscopes, and exoscopes (Table 1) [8,53].

Prominent compounds used in FGS include 5-ALA, fluorescein sodium (FS), and indocyanine green (ICG), all of which have demonstrated efficacy in delineating malignant tissue from healthy brain parenchyma. 5-ALA is a pro-drug that accumulates in tumor cells and is converted to fluorescent metabolite protoporphyrin IX (PpIX), which emits violet-red fluorescence under blue light, enabling the surgeon to visually distinguish malignant tissue [54]. The uptake of 5-ALA by the tumor microenvironment permits high diagnostic accuracy, sensitivity, and specificity, of high-grade gliomas (HGGs). FS, a fluorescent dye, is used extensively in ophthalmology and recently has gained significance in neuro-oncologic surgery [55,56]. Utilized for tumor visualization, FS accumulates in extracellular spaces where the blood–brain barrier (BBB) is disrupted [57]. With excitation at 460–500 nm, it emits green fluorescence at 540–690 nm [58]. Administered during anesthesia, FS aids in differentiating tumor tissue and is visible up to 4 h after administration [56]. Though effective, with sensitivities of 82–94% and specificities of 90–91% for HGG visualization, FS is not limited only to tumor tissue [55,56,57,58]. Dual labeling with 5-ALA may improve visualization, and further research is essential for optimal dosage and administration timing [56,59,60]. ICG is a compound that has traditionally been used for intraoperative video angiography for cerebrovascular surgery and has recently been described for intraoperative visualization of brain tumors [61,62]. ICG relies on the disrupted BBB for accumulation in brain tumors and is administered systemically over 24 h prior to surgery [63]. ICG emits fluorescence in the near-infrared range and is visualized with modified visualization devices. A recent study found that the second window ICG technique demonstrates highly sensitive detection of HGG tissue in real time [64]. They found that near-infrared imaging demonstrated a 91% correlation with gadolinium enhancement in post-surgical MRI scans, detecting residual enhancements as minute as 0.3 cm^3^, while a lack of near-infrared signals post-surgery was strongly linked to complete tumor removal, as confirmed by subsequent MRI (*p* < 0.0001).

The first and only randomized study of FGS demonstrated increased extent of tumor resection and improved progression-free survival rates [8]. New targeted fluorophores can bind to tumor-specific markers such as the epidermal growth factor receptor (EGFR) and other peptides [65,66,67,68]. 5-ALA has been the most exhaustively studied FGS agent for HGGs and most recently meningiomas due to its robust red fluorescence in the tumor bulk and predictive value for delineating tumor tissue versus surrounding brain parenchyma [69,70]. 5-ALA is the only FDA-approved agent for use during glioma surgery [71]. Correlations have also been observed between fluorescence intensity and histological grading, suggesting an ability to approximate tumor grade by fluorescent signal [6,54,69,70]. Despite its promising advantages, the use of 5-ALA comes with certain drawbacks. These include complications like photosensitivity [72]. Additionally, 5-ALA is costly and demands the use of specialized surgical visualization systems for conducting fluorescence-guided surgery (FGS) [73].

The integration of FGS into surgical loupes, microscopes, and exoscopes presents a synergistic advantage [45,74,75,76]. Recent exoscopes, equipped with light filters for 5-ALA, fluorescein, and ICG, enhance the visualization of HGGs, thereby contributing to safer and more efficient surgical procedures [40,77]. Simultaneously, the utilization of FGS within microscopes and exoscopes also yields improved resection results and patient outcomes [45,74]. Nevertheless, there are potential challenges to overcome. In addition to its restricted utility in low-grade gliomas and the low sensitivity and specificity for infiltrating tumor cells, the fluorescence intensity varies, requiring a careful interpretation of visual cues [78]. The process demands proficiency and experience to accurately distinguish between fluorescing tumor tissue and non-tumorous tissue [53,74,78].

New methods of FGS are now being studied that may permit more effective delineation of tumor tissue from the surrounding parenchyma [79]. Fluorescence lifetime imaging (FLIM) utilizes time-gated intensified cameras to visualize nicotinamide adenine dinucleotide (NADH, which is more highly expressed in tumor cells relative to normal brain tissue) and/or 5-ALA induced PpIX [79,80]. FLIM has showcased its potential to highlight areas of subtle 5-ALA fluorescence and to increase the ability to differentiate tumor cells from normal brain tissue when used to detect NADH in addition to PpIX [79]. 

Integrating fluorescence endoscopy into the spectrum of FGS offers notable benefits, especially in tackling deep-seated and elusive brain tumors. Tamura et al. [81] have reported enhanced PpIX visualization for biopsies, with a custom endoscope later employed by Potapov et al. [82]. For a comprehensive, less invasive postsurgical cavity inspection in GBM surgery. A scanning fiber endoscope identified sub-threshold PpIX fluorescence near infiltrative glioma margins, expanding upon standard wide-field operating microscope capabilities [83]. This compact, high-resolution technology promises more precision-oriented and safer neurosurgical approaches.

## 3. Intraoperative Handheld Visualization and Diagnostic Techniques

### 3.1. Raman Spectroscopy

Raman spectroscopy (RS) is a newer, innovative analytical technique that is rapidly gaining traction in neurosurgical oncology (Table 1) [15,84,85]. RS works by shining a monochromatic light, usually from a laser, onto a sample and measuring the scattering of light as it interacts with the molecules in the sample [15,84]. The scattered light undergoes a shift in energy levels, which is unique to the molecular composition and structure of the sample. This results in a spectrum that can be analyzed to provide detailed information about the chemical composition of the tissue.

The unique strength of RS lies in its ability to deliver real-time, high-resolution, and nondestructive biochemical analyses of tissues at a molecular level, thereby distinguishing tumor cells from healthy brain tissue with remarkable accuracy [15]. This characteristic is of utmost importance in glioma surgery, where differentiating neoplastic from healthy tissue is critical yet challenging [15]. Additionally, RS offers the potential for an objective, automated, and real-time feedback system, reducing the dependence on the surgeon’s subjective visual interpretation during intraoperative decision-making [86]. Recent iterations of RS have demonstrated remarkable sensitivity and specificity, which can meaningfully improve intraoperative decision-making based on rapid pathological interpretation and real-time analysis [86,87,88]. 

RS has rapidly translated from the research to the clinical setting. The work by Jermyn et al. was a seminal breakthrough, bringing a hand-held RS probe into the operating room with striking outcomes—achievements that have since been commercialized [89]. The reported 93% sensitivity and 91% specificity illustrate the potential for widespread clinical adoption. This success has been echoed in other innovative applications, such as an imaging needle for intraoperative blood vessel detection and a dual-modal system combining surface enhanced Raman scattering (SERS), which involves the amplification of Raman signals using metal nanoparticles, and optoacoustic tomography, which uses the generation of ultrasound waves through light absorption to create detailed images, for tumor delineation [85,90,91,92]. Coherent anti-Stokes Raman scattering (CARS), a nonlinear technique sensitive to molecular vibrations, has also shown promising results, particularly in distinguishing healthy cells from cancerous ones [93]. This is achieved by using two laser beams—pump and Stokes beams—that are tuned to match the energy difference between the ground and excited vibrational states of the target molecules. The development of this technique has been further enhanced by the inclusion of stimulated Raman Scattering (SRS) [94,95]. SRS is another advanced spectroscopic method, which also uses two laser beams but in a slightly different manner to generate a signal that is directly proportional to the concentration of the target molecules. This advanced spectroscopic method has demonstrated its capability to emulate the conventional hematoxylin and eosin (H&E) staining technique with a diagnostic accuracy exceeding 92%. Such advancements contribute an additional layer of diagnostic proficiency to optical technologies. Recent studies have highlighted the ability of SERS in differentiating tumor types, while SRS has been shown to effectively identify human brain tumor infiltration [86,96]. Desroches et al. [97] provided another leap forward by developing a core needle biopsy probe incorporated with a navigation-guided fiber optic Raman probe, enhancing in situ surgical capabilities. Their handheld spectroscopy system demonstrated robust sensitivity and specificity rates of 80% and 90%, respectively, for the intraoperative detection of malignancies. Concurrent advancements in data analysis machine learning (ML) methods, such as principal component analysis, classical least square fitting, partial least square, and linear discriminant analysis, continue to expand the possibilities of Raman-based techniques in brain tumor surgery [86,98,99].

Despite the promising advances in RS in brain tumor surgery, significant challenges linger, constraining its clinical integration. Raman techniques frequently suffer from weak signal intensity, requiring considerable effort to enhance the signal-to-noise ratio [86,100]. Data acquisition and processing times can complicate their real-time applicability in clinical settings [101]. However, semiautomated methods are currently in development to streamline RS measurements for the detection of brain tumors in real-time during surgery. 

### 3.2. Confocal Microscopy

Confocal microscopy (CM) is another advanced optical imaging tool that has handheld applications with a foot switch and plays a pivotal role in enhancing the precision of brain tumor resections (Table 1) [54,102]. CM functions by employing spatial filters, such as pinholes and slits, to effectively eliminate out-of-focus and multiply scattered background light, thereby enabling optical sectioning microscopy [103]. Consequently, it can generate high-contrast images and offer micron-scale spatial resolution, reaching up to approximately 100 μm imaging depth within tissue, allowing for the visualization of structures in three-dimensional volume [104,105]. Notably, these features contribute to the efficacy of CM in distinguishing between healthy and cancerous brain tissues [104]. Additionally, the technique capitalizes on the detection of fluorescence markers, like PpIX, for accurate visualization and delineation of low-grade gliomas [105]. 

Confocal laser endomicroscopy (CLE) is a type of CM that has recently demonstrated promising advancements in intraoperative brain tumor surgery with handheld applications. Höhne et al. implemented CLE in surgical protocols, administering 5 mg/kg of sodium fluorescein (SF) to a cohort of 12 patients [106]. They found the procedure beneficial in providing high-quality visualization of fine structures and for presenting concealed anatomical detail, indicating SF’s potential as a reliable contrast agent. Moreover, in 2022, Abramov et al. explored CLE’s in vivo feasibility for brain tumor surgeries, reporting high diagnostic accuracy and quick image acquisition [107].

Despite its potential, CM in brain tumor surgery is challenged by several issues. Commercial intraoperative confocal microscopes currently rely on fluorescein for visualization of tumor tissues and are not fully optimized for PpIX fluorescence visualization. Traditional single-axis probes often suffer from motion artifacts due to slow frame rates [108,109]. Current visualization of tissues is based on black and white imaging and these devices also lack adjustable imaging depth. Thus, while promising, CM requires further refinement for improved clinical efficacy [110].

## 4. Conventional Imaging Techniques for Intraoperative Tumor Resection

Traditional imaging technologies such as iUS, iMRI, and iCT have greatly enhanced suprasellar imaging visualization and the extent of tumor resection while also helping navigate complex anatomical changes (Table 1) [1,5]. An optimal approach may involve the integration of cellular/subcellular imaging for diagnostic specificity with supracellular imaging techniques for broader resection evaluation. This could potentially increase the precision and effectiveness of brain tumor surgeries.

Since its introduction in the 1980s, iCT has witnessed significant improvements in overcoming initial challenges of image quality and equipment-related artifacts [111,112,113]. Although not used as commonly, it offers rapid image acquisition, cost efficiency, and better workflow compatibility [2]. iCT’s capability to capture images while the patient’s head is secured in a head clamp is an undeniable asset. This feature facilitates updating the neuronavigation system to account for brain shift and allows for vascular imaging [111]. The technological evolution has spurred the creation of automated registration techniques, thereby reducing the average target registration error to less than 1 mm [114].

iUS uses the principle of piezoelectricity to generate real-time images of brain structures [115]. Presenting as the most cost-effective intraoperative imaging, iUS provides immediate feedback, facilitating tumor boundary localization and brain anatomy changes, thus enhancing tumor resection precision [3,116]. However, the complex echo patterns and low image quality require advanced interpretation and operational skills [117,118]. Newer technologies such as Doppler ultrasonography, contrast-enhanced iUS (CEUS), and elastography have improved visualization and tumor boundary delineation, brain tumor vascularization assessment, and tumor grade differentiation [119,120,121]. 

iMRI is a new adaptation of an existing diagnostic technology, offering superior resolution and tissue differentiation capabilities while providing a better extent of resection compared to other modalities [117,118]. Similar to iUS, iMRI counters the ‘brain shift’ phenomenon during surgery, crucial in maintaining stereotactic navigation accuracy and maximizing tumor extent of resection [6,122,123,124,125]. However, widespread adoption of iMRI is still limited due to its clinical criteria, high costs, length of acquisition, and operating room infrastructure logistic needs [30,124]. Future directions in iMRI aim to focus on enhancing image quality, workflow efficiency, and cost-effectiveness with low-field units while also integrating other imaging modalities [117,118]. However, a recent study by Roder et al. [126] compared iMRI and 5-ALA in glioblastoma surgery. The study found that both iMRI and 5-ALA were comparable in achieving complete resections, defined as residual tumors ≤ 0.175 cm^3^ (81% for iMRI vs. 78% for 5-ALA; *p* = 0.79).

The landscape of intraoperative imaging is constantly evolving, fueled by converging technological forces that are revolutionizing neurosurgical procedures [18,33,63]. This transformation in neurosurgical imaging is being driven by three key developments: the integration of multiple real-time imaging modalities, the incorporation of augmented reality (AR)/virtual reality (VR), and the incorporation of artificial intelligence (AI) and ML [63,127,128,129,130]. The integration of multiple modalities in intraoperative imaging is proving instrumental in advancing neurosurgical innovation. This integrated approach is the result of a growing recognition that no single modality holds the answer to all clinical questions [7,63,131,132]. 

AR and VR are poised to revolutionize surgical planning, navigation, and surgical execution [129]. These technologies overlay digital information onto the physical world (AR) or create entirely simulated environments (VR), thereby transforming neurosurgical procedures [129,130,133]. Sun et al. [129] highlighted the potential of AR and VR in neurosurgical operations. Their study involved 79 glioma patients and 55 control subjects and demonstrated that utilizing functional neuronavigation and intraoperative MRI enables tailored and optimized surgery. The AR group showed significantly higher complete resection rates (69.6% vs. 36.4%) and average extent of resection (95.2% vs. 84.9%) compared to the control group, with statistical significance (*p* < 0.01). The preservation of neural functions was also superior in the AR group at 2 weeks and 3 months postoperatively. These research findings highlight the effective role of AR in enhancing accuracy and advancing patient outcomes during the surgical removal of tumors in eloquent areas of the brain. The use of AR technology is not limited to these procedures; it also plays a significant role in providing guidance during intraoperative navigation in endoscopic skull base surgeries. Pennachietti and colleagues discovered that when AR is incorporated into endoscopic skull base approaches, it helped accurately target neurological lesions and determine the intraoperative extent of a tumor [134]. Zeiger and his team further demonstrated that AR can be beneficial in identifying the boundaries of standard bony structures during skull-base surgeries, such as delineating the relation of the anterior clinoid to the optic nerve and internal carotid artery [135]. Nevertheless, it is significant to highlight that the majority of these studies on surgical resections guided by AR have primarily relied on pseudo-AR techniques, involving overlays inserted into the lenses of microscopes equipped for AR functionality.

Finally, the future of intraoperative imaging in neurosurgical oncology is guided by the synergistic incorporation of AI and ML [127,128,136]. The capability of these systems to refine the delineation of tumor and healthy tissues is pivotal to the success of tumor diagnostics and surgical resections, promising enhancements in both surgical precision and patient outcomes [128,137]. An exemplar of this synergy is the integration of AI with FGS, a vital technology in differentiating tumor tissue during brain tumor surgery [137,138]. Multiple studies illustrated the utility of AI for real-time intraoperative cytological diagnosis of CNS tumors and training a deep learning model on a variety of brain lesions [127,128,136]. In this context, ‘patch-level classification’ refers to the AI system’s ability to diagnose tumors based on small, localized areas or ‘patches’ of the scanned images, while ‘patient-level classification’ indicates the system’s ability to integrate these individual diagnoses into a comprehensive understanding for each patient. Remarkable diagnostic accuracies of 95% and 97% were achieved in the patch-level classification and patient-level classification tasks, respectively, emphasizing the potential of AI and its future trajectory in the intraoperative diagnosis of brain tumors [128]. The application of AI and ML in managing the increasing complexity and volume of data from multimodality imaging systems cannot be overstated [127,128,136]. These tools have the potential to improve image quality, detect subtle patterns that might be overlooked by human perception, and provide predictive insights based on preoperative imaging.

The future of intraoperative imaging is one of convergence and augmentation. As these technologies merge, allowing surgeons to harness the advantages of each visualization modality simultaneously in real time in the operating room, we find that neurosurgical oncology stands on the brink of transformation. The implementation of these advancements is contingent upon continued collaborative efforts among clinicians, engineers, physicists, and scientists to navigate challenges and maximize the potential of these technologies.

## 5. Conclusions

Advancements in intraoperative imaging and optics—such as the exoscope, FGS, RS, and CM—are propelling forward the field of brain tumor surgery. Despite the progress, several challenges persist, including steep learning curves and difficulties in image interpretation. We advocate for an integrative approach that synergizes subcellular imaging and diagnostics with supracellular imaging modalities. When combined with intraoperative techniques like fluorescence-guided surgery, this comprehensive strategy has the potential to enhance tissue differentiation, thereby improving surgical outcomes for patients. Future trends look toward incorporating AI and ML, integrating various imaging modalities, and applying AR and VR. Sustained interdisciplinary collaboration is essential for unlocking the full potential of these innovative technologies, all geared toward the ultimate goal of enhancing the precision and effectiveness of surgical interventions for brain tumor patients.

## Figures and Tables

**Table 1:** A Summary of the Intraoperative Imaging Modalities Used for Brain Tumor Resection.

Modality	Concept Description	Advantages	Limitations
Intraoperative Ultrasound (iUS)	Utilizes high-frequency sound waves to create images of the brain during surgery	Provides dynamic feedback; enhances tumor localization	Limited by operator experience; may not be effective for all tumor types
Intraoperative Magnetic Resonance Imaging (iMRI)	Utilizes magnetic fields and radio waves to create detailed images of the brain during surgery	High-resolution imaging; detect brain shift	Requires significant infrastructure; may prolong surgery time
Intraoperative Computed Tomography (iCT)	Utilizes X-ray technology to create cross-sectional images of the brain during surgery	Rapid image acquisition; Seamless incorporation into surgery	Exposure to ionizing radiation; lower soft tissue contrast compared to MRI
Surgical Microscope	An optical instrument with high magnification used during brain tumor surgery for precise visualization	High-definition visualization; differentiation between healthy tissue and tumor; facilitates maximal safe resection; can record surgical procedures in high-definition	Restricted field of view; limited maneuverability due to bulk and weight; operator fatigue due to ergonomics
Exoscope	A high-definition camera that offers a panoramic view of the surgical area	Improved magnification and illumination; better depth of field; enhances ergonomics for surgeons	Potential learning curve for new users; cost of integration into the surgical workflow
Fluorescence-Guided Surgery (FGS)	Utilizes fluorescent agents to delineate tumor tissue during surgery, providing real-time intraoperative tumor visualization	Real-time visualization; facilitates maximal safe tumor resection	Limited by the availability of fluorescent agents; may not be effective for all tumor types
Raman Spectroscopy	Uses monochromatic light for real-time, high-resolution biochemical tissue analysis at molecular level	Distinguishes tumor cells from healthy brain tissue with high accuracy; objective and automated feedback	Weak signal intensity; challenges in data acquisition and processing times for real-time applicability
Confocal Microscopy	Uses spatial filters for high-contrast, micron-scale resolution imaging	High-contrast and detailed images; visualization of structures in three-dimensional volume; employs detection of fluorescence markers for tumor visualization	Motion artifacts due to slow frame rates; relies on specific agents; lacks adjustable imaging depth
